# Currently favored sampling practices for tumor sequencing can produce optimal results in the clinical setting

**DOI:** 10.1038/s41598-020-71382-3

**Published:** 2020-09-01

**Authors:** Lőrinc S. Pongor, Gyöngyi Munkácsy, Ildikó Vereczkey, Imre Pete, Balázs Győrffy

**Affiliations:** 1 Department of Bioinformatics, Semmelweis University, Budapest, Hungary; 2 Momentum Cancer Biomarker Research Group, Institute of Enzymology, Research Center for Natural Sciences, Budapest, Hungary; 3 National Institute of Oncology, Budapest, Hungary; 4 2nd Department of Paediatrics, Semmelweis University, Budapest, Hungary

**Keywords:** Cancer genetics, Cancer genetics, Cancer, Computational biology and bioinformatics, Genetics, Oncology

## Abstract

Tumor heterogeneity is a consequence of clonal evolution, resulting in a fractal-like architecture with spatially separated main clones, sub-clones and single-cells. As sequencing an entire tumor is not feasible, we ask the question whether there is an optimal clinical sampling strategy that can handle heterogeneity and hypermutations? Here, we tested the effect of sample size, pooling strategy as well as sequencing depth using whole-exome sequencing of ovarian tumor specimens paired with normal blood samples. Our study has an emphasis on clinical application—hence we compared single biopsy, combined local biopsies and combined multi-regional biopsies. Our results show that sequencing from spatially neighboring regions show similar genetic compositions, with few private mutations. Pooling samples from multiple distinct regions of the primary tumor did not increase the overall number of identified mutations but may increase the robustness of detecting clonal mutations. Hypermutating tumors are a special case, since increasing sample size can easily dilute sub-clonal private mutations below detection thresholds. In summary, we compared the effects of sampling strategies (single biopsy, multiple local samples, pooled global sample) on mutation detection by next generation sequencing. In view of the limitations of present tools and technologies, only one sequencing run per sample combined with high coverage (100–300 ×) sequencing is affordable and practical, regardless of the number of samples taken from the same patient.

## Introduction

Multi-region sequencing has shown that distinct regions of a tumor have unique sets of clonal, sub-clonal as well as private mutations^1^ rendering the estimation of tumor heterogeneity a challenging task in terms of experimental design^2^. Of note, both passenger and driver genetic changes can be distributed heterogeneously within a single tumor^3^. This phenomenon is of high clinical relevance since an analysis of over 3,000 tumors in nine different cancer entities revealed that high levels of intratumoral heterogeneity was unequivocally linked to worse survival^4^.

Darwinian evolution of tumor cells means that higher heterogeneity leads to a higher chance of having a clone with inherent resistance to an applied systemic treatment. When administering a targeted therapy regime, different clones can use diverse mechanisms to confer resistance—and at the same time, multiple sites of the same tumor frequently show a convergent loss of the same suppressor as a tool to establish resistance^5^.

In ovarian cancer, testing for germline and somatic BRCA1/2 mutations helps to identify subgroups of patients where utilization of the PARP inhibitors olaparib or rucaparib can have significant therapeutic advantage^6^. Olaparib has been approved by the FDA for BRCA1/2 carrier advanced HER2-negative breast cancers, while PARPs inhibitors are also under phase III trial for multiple cancer types, such as ovarian, prostate, pancreatic, and lung cancers (www.clinicaltrials.gov). Recently, it has been shown that HR-deficiency can be identified based on genomic characteristics of tumors, factoring chromosomal instability, rearrangements, and somatic mutation signatures, which in turn are influenced by the tumor site (e.g. primary or metastasis) and by sampling strategy.

Taken together, cancer heterogeneity significantly affects systemic therapy and is a major obstacle to cancer control. Generally, only a single sample of the tumor is analyzed by sequencing in personalized medicine—this means that spatial heterogeneity cannot be accounted for. Thus, the question arises of how to execute the right sampling—what is an optimal size? Could we identify spatially separated mutations by combining multiple regions? Here we scrutinize how different sampling techniques affect mutation detection in a set of ovarian tumors. Our data show that high coverage sequencing of single or pooled samples appears to be an optimal strategy for clinical settings.

## Results

### Genomic characteristics

**Figure 1:**
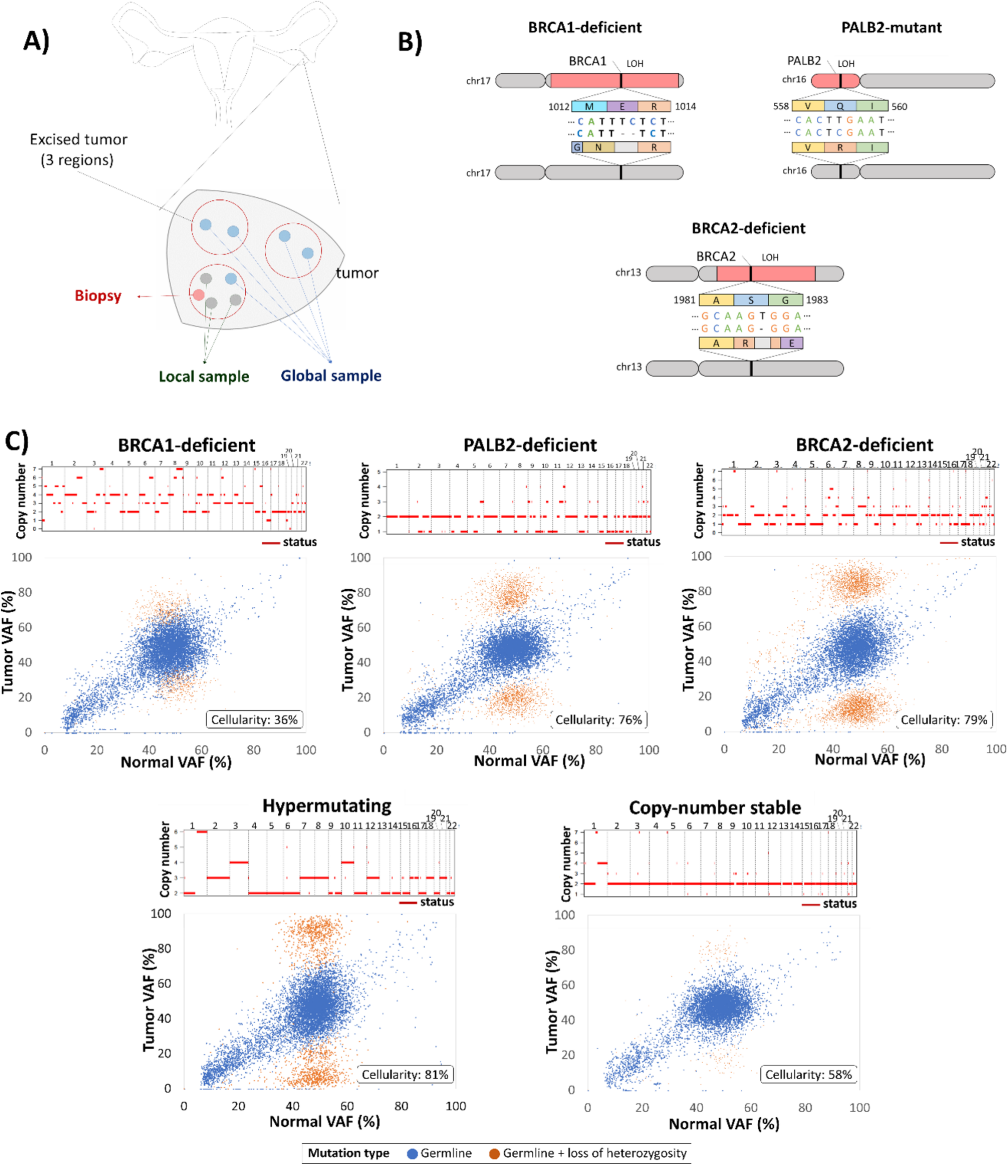
Sample isolation and copy-number variation characteristics. (**A**) Sample isolation strategy from tumor regions. Three portions of each tumor were obtained, from which DNA isolation was performed as a biopsy sample, local sample (directly surrounding the biopsy), and global sample (all three regions merged). (**B**) Identified heterozygous germline mutations affecting homologous recombination with somatic loss of heterozygosity. (**C**) Copy number alterations (upper) and mutation frequency shift of variants affected by somatic loss of heterozygosity (lower).

We picked five different setups for in-depth analysis. Single tumor region, local sample, and global sample were taken and analyze from five ovarian cancer patients (see Materials and Methods, Fig. 1A). We selected three patients with a germline mutation affecting genes in the BRCA pathway (Fig. 1B) termed as “HR-deficient” or “BRCA-deficient” patients. The first patient termed as “BRCA1-deficient” harbored a frameshift BRCA1 mutation (rs397507208), that was covered by somatic loss of heterozygosity event (*p* = 1.5E−03). This tumor also had a heterozygous frame-shift variant in BRCA2 that was not covered by a LOH event. The second patient termed as “BRCA2-deficient” had a pathogenic frameshift mutation in the BRCA2 gene (rs80359550), which was covered by a LOH event (*p* = 6.5E−05). A third patient (hence on termed as “PALB2-mutant”) harbored a mismatch mutation and somatic LOH (*p* = 1.7E−08) in the PALB2 gene, which was previously associated with breast cancer^7^.

Copy-number alterations and loss of heterozygosity (LOH) events were very dissimilar among the patients (Supplementary Table 1). We identified a “copy-number stable” patient (Fig. 1C), with one partial amplification on the first chromosome, and one patient with multiple amplifications affecting entire chromosomes (Fig. 1C) with three-fold more somatic mutations (hence termed as “hypermutating”). The HR-deficient tumors displayed multiple events on smaller chromosome segments (Fig. 1C). One patient (“copy number stable”) displayed far fewer events than the remaining patients did (n = 250 in the “copy number stable” vs. n = 1,015–5,050 in other samples).

**Figure 2:**
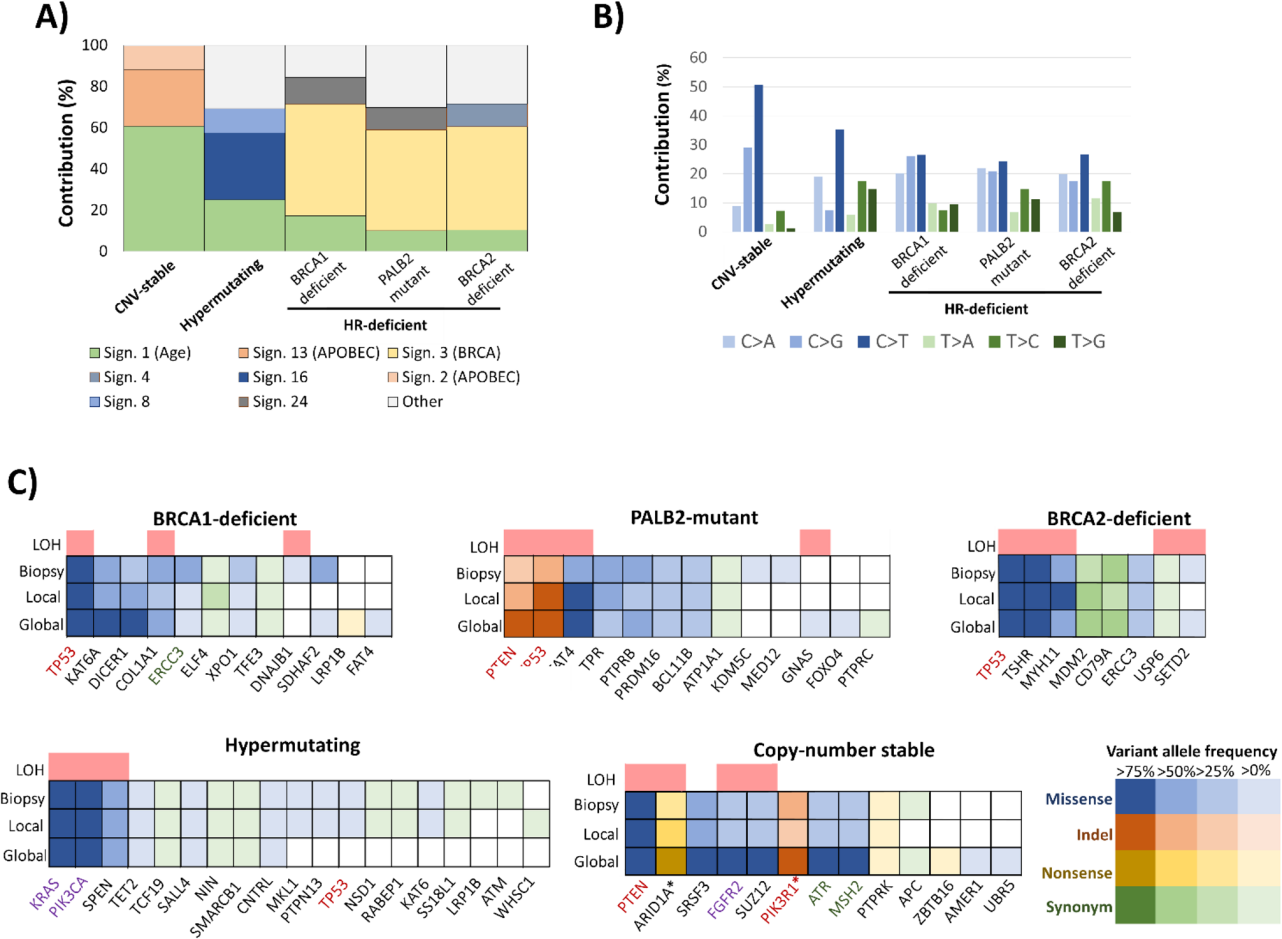
Somatic mutation signatures and affected cancer genes. (**A**) Identified mutation signatures of somatic mutations and (**B**) base substitution spectrum. HR-deficient tumors displayed the BRCA-deficiency associated “signature 3”. (**C**) Cancer consensus genes with identified somatic mutations.

Somatic mutation patterns in HR-deficient tumors displayed the BRCA-deficiency associated Signature 3 (Fig. 2A), where base substitution proportions were more evenly distributed across the four nucleotides (Fig. 2B). We identified signatures 2 and 13 in the copy-number stable tumor, which are associated to the activity of the APOBEC cytidine deaminases family and highly increased C > T somatic substitutions. In case of the hypermutating tumor, two signatures with unknown origin were found (Supplementary Table 2).

Of note, when investigating mutations in key oncogenes and tumor suppressor genes (Fig. 2C), HR-deficient tumors were driven by DNA-repair defect paired with somatic TP53 mutations. In case of the hypermutating tumor, we identified the two most common KRAS and PIK3CA activating mutations. The copy-number stable tumor contained multiple mutations affecting key genes in the PI3K pathway, such as PTEN, FGFR2 and two deleterious mutations in the PIK3R1 gene.

### Mutation frequencies

**Figure 3:**
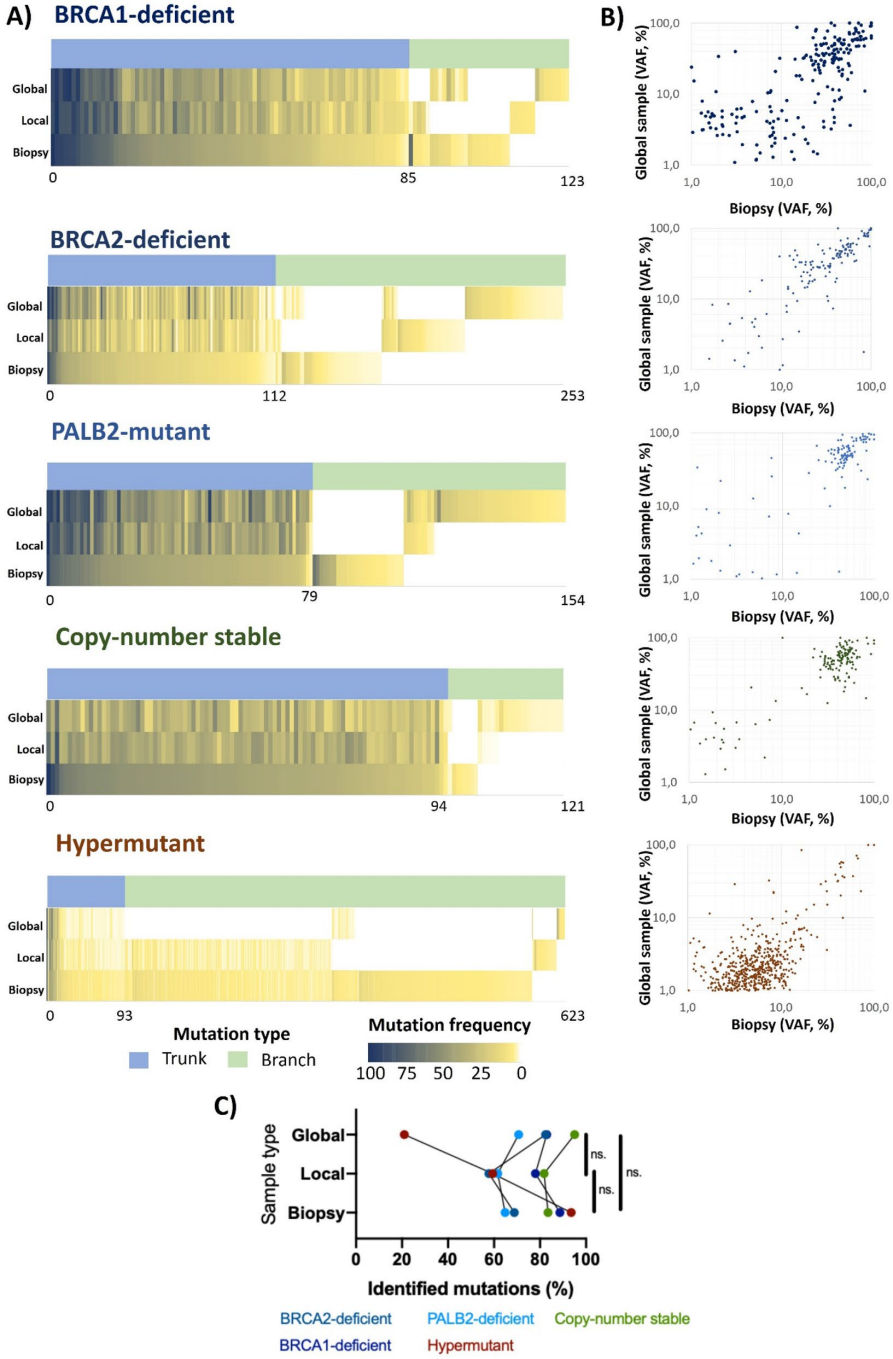
Effect of sampling strategy on detectable somatic mutations. (**A**) Distribution of somatic mutations. Heat map indicates presence (non-white), and mutation frequency from 100% (dark blue) to 1% (light yellow). Trunk (common in all samples) and branch (non-common) mutations are represented with blue or green bars. (**B**) Comparison of variant allele frequency between biopsy and global samples. (**C**) Percentage of mutations identified with mutation calling in each region from the tumors’ combined mutation calls.

When evaluating sequencing outcomes related to different sampling strategies, high frequency clonal mutations were common across all samples, while low-frequency sub-clonal mutations displayed clonal evolution patterns (Fig. 3A). The number of trunk mutations (shared in every region) spanned between 79 and 112 in all patients. While in terms of absolute value, most mutations were identified in the biopsy sample of the hypermutating tumor (n = 583), only 15.9% of these were trunk mutations (Supplemental Fig. 1A). The proportion of trunk mutations increased as the sample size increased and reached 71.4% of mutations in the global sample, comparable to proportions detected in the other samples. This shift in mutation proportions in the hypermutating tumor may be caused by the dilution of sub-clones with increasing tumor size. The highest level of homogeneity was found in the copy-number stable tumor, where > 81.7% of mutations in any sample were trunk mutations.

By comparing mutation variant allele frequencies (VAFs) between samples, we identified different evolution patterns. In case of the BRCA1/2 mutant (HR-deficient) tumors, somatic mutation VAFs were similar between samples and evenly distributed (Fig. 3B). The copy-number stable and PALB2-mutant tumors harbored predominantly high frequency mutations, possibly hinting on a “big bang” model of evolution^8^. In case of the hypermutating tumor, high frequency clonal mutations were similar between samples, while sub-clonal mutations had higher VAFs in the biopsy sample (Fig. 3B).

In the PALB2-deficient, BRCA2-deficient, and copy-number stable tumors the global sample identified slightly higher proportions of mutations, while in the BRCA1 and hypermutating tumors the biopsy samples covered higher proportions of mutations (Fig. 3C). However, there were no statistically significant differences in the proportion of identified mutations when performing pairwise comparisons between the biopsy, local, and global samples were computed (Fig. 3C).

Cases with available metastatic samples can be used to simulate sample pooling. For this analysis, we utilized data from a clear renal cell carcinoma (ccRCC) multi-region sequencing study^1^. Gerlinger et al. uncovered the branched evolution of ccRCC by sequencing multiple distinct regions of the same tumor. While some mutations were shared among distinct regions of the tumor (trunk), other branch mutations were shared across samples, and there were also several sample specific (private) mutations. We utilized samples from their study because primary and metastatic samples were available, and because ccRCC tumors show similar tumor evolution patterns as ovarian tumors^9–11^. Our analysis focused on the possibility of pooling samples to capture a wider set of variants without the need to sequence multiple samples from each tumor. We modelled the effects of sample size by sampling from the primary and metastatic sites, changing the composition ratios of the sites and coverage depths. Through combination of data derived from primary and metastatic samples we modelled the effect of high intra-tumor heterogeneity on sampling and mutation composition shifts (Supplemental Fig. 1B). From the in silico results we uncovered that most clonal branch mutations of the minor sample could be identified when proportion reached 20% composition, where 93% of all clonal mutations (including both trunk and region specific) were identified. Most clonal mutations (> 95%) were identified when the composition of the two samples ranged between 30 and 60%. At the same time, there was no composition generating a clear maximum in mutation detection. We have also seen this when we examined the effect of sequencing coverage on mutation calling. When coverage reached the original sequencing coverage (onefold), 92% of clonal mutations were identified, but interestingly only 10% of sub-clonal mutations. As coverage increased, there was no radical increase in identified clonal mutations, while the percentage of identified sub-clonal mutations increased in a linear fashion until coverages reached ~ 300 × (Supplemental Fig. 1C).

## Discussion

Intra-tumor heterogeneity remains a major factor influencing resistance against systemic anticancer therapies as well as metastatic progression of the tumor. Previous works suggest that the seeds of later clonal diversity are typically present very early in tumorigenesis, and intra-tumor heterogeneity is pervasive thereafter^8,12^. Thus, it stands to reason that limits of detection may be a more pressing issue than extent or spread of and intra-tumor heterogeneity by the time a tumor presents clinically. Diversity of detectable clones at presentation may be more clinically and therapeutically relevant than their extent or spread^13^. While these studies provide insight into heterogeneity, they cannot serve as models for clinical sequencing as this later usually involves sequencing from only one biopsy due to time and cost limitations.

The utilized sampling method can fundamentally determine sequencing results. Here, we analyzed the effect of the sequenced tumor size on the detected mutations using next-generation sequencing on a representative but genetically diverse set of ovarian cancer patients. Our findings suggest that size of the sequenced sample does not significantly affect detected composition in case of non-hypermutating tumors. In hypermutating tumors, size increase drastically reduces the detected mutations. This phenomenon roots in the dilution of sub-clones, rendering the tumor seemingly more homogenous due to detection thresholds in current next-generation sequencing runs. Thus, increasing sample size can deteriorate overall mutation detection rate and could thereby even completely cloud hypermutating tumors.

One potential concern can arise from the extreme structural variation observed in high-grade serous ovarian carcinomas tumors. As previously noted^14^, inversions detectable via WGS but not, a priori, by WES constitute a clinically relevant subtype of ovarian carcinoma. High-grade serous ovarian carcinomas are notorious for developing resistance to PARP inhibitors at progression^15^ and this appears to frequently arise from either activating fusions of ABCB1 or functionally revertant variants arising in BRCA and Fanconi anemia genes. To the extent that subclonal structural variants represent actionable targets (e.g. NTRK fusions), this could represent a weakness in 100 × –300 × WES as a clinical standard. Nevertheless, one can assume that clinical sequencing will eventually move towards either ultra-deep (500 × –1,000 ×) long-read panel sequencing and/or ~ 100 × WGS, obviating this issue.

Our results also suggest that combining two samples (i.e. primary and metastatic) paired with molecular barcoding could enable a > 95% detection of mutations from both samples. In addition, standard sequencing coverage (~ 70 ×) enable reliable clonal mutation detection, while sub-clonal detection requires higher coverage due to low cellular prevalence and technical deviations of next-generation sequencing^16^. Theoretically, by increasing coverage, we should be able to identify all mutations within a sample^17^. It seems quite logical that biological sample size (patients profiled) is of greater clinical relevance than technical sample size—a recent work supports this notion for rare tumors driven by structural variants^18^. However, to achieve reliable results, instead of utilizing simple hard cutoffs and relative frequencies, mutation callers have to be optimized for high coverage sequencing scenarios^17,19^.

How can our study give implications to derive treatment decisions? Minor clones have a low priority for targeted therapy as we can only expect the founder mutations with high mutation rate to be present in all sub-clones within a single tumor. We have observed concordant high variant allele frequencies when comparing global samples to biopsy samples—this favors a single biopsy which is at present more feasible due to less work and lower cost of the sequencing. These results also provide explanation why current diagnostic assays utilizing a single tumor sample (including multigene tests like the Oncotype DX, Endopredict, Mammaprint, or BRCA sequencing) can accurately predict treatment response and survival in most of the patients despite the presence of intra-tumor heterogeneity^20^.

In hypermutating tumors, size increase drastically reduces the detected mutations. This phenomenon roots in the dilution of sub-clones, rendering the tumor seemingly more homogenous due to detection thresholds in current next-generation sequencing runs. Thus, increasing sample size can deteriorate overall mutation detection rate and could thereby even completely cloud hypermutating tumors. The fact that increasing sample size in hypermutating tumors reduced the number of mutations detected suggests that the local and global samples may have more mutations that were not detected using available methods. Additional analyses will be needed to exactly determine the optimal sample processing strategy in hypermutating tumors.

Finally we have to note that there is still a substantial number of branch mutations that are missed, whether using biopsy, local or global samples. This suggests that while all three strategies of tumor sampling may be suitable in the clinical setting, they may not be sufficient for studying tumor heterogeneity.

In summary, we compared the effects of sampling strategies (single biopsy, multiple local samples, pooled global sample) on mutation detection by next generation sequencing. In non-hypermutating tumors, an increase in sample size improves detection of sub-clonal mutations. When combining primary and metastatic samples, an increase in sequencing depth only affected sub-clonal mutational detection. Our results suggest that, considering the available technological and analytical tools, currently favored sampling practices can produce optimal results in the clinical setting. In other words, high coverage sequencing of a single sample can account for the problem of intratumor heterogeneity.

## Material and methods

### Tumor sample collection

We collected fifteen frozen tissue samples at the National Institute of Oncology (Budapest, Hungary) from five high-grade ovarian cancer patients. None of the selected patients received neo-adjuvant therapy, which could influence mutational composition of the tumor samples (Supplementary Table 3). Ethical approval for the study was granted by the Ethics Committee of the National Institute of Oncology (Budapest, Hungary) under the number OOI ALT-9444-1/2013/59. Using multiple tumor regions within a single primary tumor, we extracted DNA in three settings: (a) a biopsy from a single tumor region, (b) a local sample deriving from three neighboring regions around the biopsy in the same tumor region, and (c) a global sample, where DNA was extracted and combined from three distinct sampling regions. In each setting, a total of three DNA samples were generated as seen in Fig. 1A. For control, DNA blood samples were collected from each patient. This study was conducted in accordance with the Declaration of Helsinki.

### DNA isolation and sequencing

We isolated DNA from the tumor and blood samples using the Qiagen DNeasy Blood and Tissue Kit following the manufacturer's protocol (Qiagen GmbH, Hilden, Germany). A volume of 200 µl was used as starting material for isolation from blood (Supplementary Table 4). Four DNA samples including three tumor samples (biopsy, local sample and global sample) and one blood sample were isolated for each patient. *Biopsy* sample was defined as a 15 mg tissue; *local sample* was a 30 mg tissue around the biopsy sample; *global sample* was a 30 mg specimen combined from the local area and two distinct areas of a biopsy sample (Fig. 1A). DNA was eluted in 100 µl DI water and isolated total DNA concentrations were validated with a NanoDrop ND-1000 spectrophotometer (BCM, Houston, TX, USA) and Qubit 3.0 Fluorometer (Thermo Fisher Scientific, USA). DNA samples were stored at − 80 °C and were sequenced on the Illumina NextSeq 500 next generation sequencing platform. Whole-exome sequencing of the normal blood and tumor samples was performed with 100 × coverage.

### NGS data processing

The *bwa mem* program^21^ was used to align reads to the human genome (GRCh38) downloaded from UCSC site (https://genome.ucsc.edu/). Aligned reads in SAM format were sorted and converted to BAM format using *samtools*^22^. Germline mutations were called using the Haplotype caller of the GATK Package^23^ in the gVCF mode, while somatic mutations were identified using the *mutect2* algorithm^23^. Somatic mutations were filtered based on the *mutect2* judgment as well as additional hard filters (at least 50 × coverage, at least 5 × mutation coverage). Joint genotyping of identified somatic mutations was performed with an in-house program written in python (github: goo.gl/WrmRQ4), where a genotype is accepted by comparing the number of mutant reads and the background noise derived from the intermediate regions using a Poisson cumulative distribution test (Supplementary Table 5). Copy-number analysis and tumor purity estimation (Supplementary Table 6) were performed using *sequenza*^24^. Identification of loss of heterozygosity (LOH) variants was performed by selecting heterozygous germline mutations with B-allele loss events determined by *sequenza*, followed by comparison of mutation frequency shift between the normal and tumor samples using Fisher’s exact test. We summarized the number of germline variants for each patient with and without LOH in the biopsy sample of the tumors for CNV segments identified by *sequenza* in Supplementary Table 7. Mutation signatures were analyzed using high quality, high coverage (> 50 × coverage, > 10 mutant reads) somatic mutations that were common across all regions with the *MutationalPatterns* package^25^, where contributions were calculated using “signatures of mutational processes in human cancer” signatures (source: https://cancer.sanger.ac.uk/cosmic/signatures).

To create somatic mutational heatmaps, we first selected variants with sufficiently high sequencing depth (50 ×), variants with at least 5 altered reads in one of the regions, and variants with at least 5% variant frequency after correction with the estimated tumor purity from *sequenza* (Supplementary Table 6). Mutation frequencies for variants were used in cases where the variant was identified by either *mutect2* or the *mcaller* (Fisher test).

### Statistical analysis

We performed a successive analysis by computationally mixing reads from two regions (primary tumor and metastatic site) of a single patient from a previously published dataset^1^. Both the primary and metastatic samples enclosed site-specific mutations, thus combining reads from these two regions generated a model of high intra-tumor heterogeneity. Mutations were defined as clonal, if the variant allele frequency (VAF) was above 10%, and sub-clonal if the VAF was less than 10%. Reads from the two regions were first combined in compositions between 10 and 90% interval at every decile with ten replicates. To better understand how coverage affects mutation calling, we combined reads from both experiments in equal proportions between 0.1 and 100 fold of the original BAM files (where onefold coverage represents coverage of the original BAM files combined). Random sampling was performed by extracting 10% of reads at a time (using a random seed) until designated percentage was reached.

### Data availability

The sequencing data is available at the European Genome-Phenome Archive via the accession number EGAS00001004200 (https://www.ebi.ac.uk/ega/studies/EGAS00001004200).

### Patient consent

We have obtained informed consent from all participants and this is noted in the manuscript.

## Supplementary information

Supplementary Figue 1.

Supplementary Table 1.

Supplementary Table 2.

Supplementary Table 3.

Supplementary Table 4.

Supplementary Table 5.

Supplementary Table 6.

Supplementary Table 7.
