# Supplementary material for: Currently favored sampling practices for tumor sequencing can produce optimal results in the clinical setting
Source: Sci Rep. 2020 Sep 1;10:14403. doi: 10.1038/s41598-020-71382-3 (PMC7463012; doi:10.1038/s41598-020-71382-3)

**Currently favored sampling practices for tumor sequencing can produce optimal results in the clinical setting**

Lőrinc S. Pongor^1,2^ - Gyöngyi Munkácsy^1,2^, Ildikó Vereczkey^3^, Imre Pete^3^, Balázs Győrffy^1,2,4^

^1^ Semmelweis University Dept. of Bioinformatics, Budapest, Hungary

^2^ Momentum Cancer Biomarker ResearchGroup, Institute of Enzymology, Research Center for Natural Sciences, Budapest, Hungary

^3^ National Institute of Oncology, Budapest, Hungary

^4^ Semmelweis University 2nd Dept. of Pediatrics, Budapest, Hungary

**Supplemental Figure 1. Effects of high intra-tumor heterogeneity on mutation composition shifts.** **A)** Samples selected for in silico analysis deriving from multi-region sequencing data. One primary (R9) and one metastatic (M1) sample was selected. **B)** Percentage of total mutations identified at any given composition of selected regions. As composition of minor sample reached 30%, majority (>95%) of all mutations could be identified. **C)** Effect of sequencing coverage on detected mutations. All mutations (red), clonal mutations (black) and sub-clonal mutations (grey).


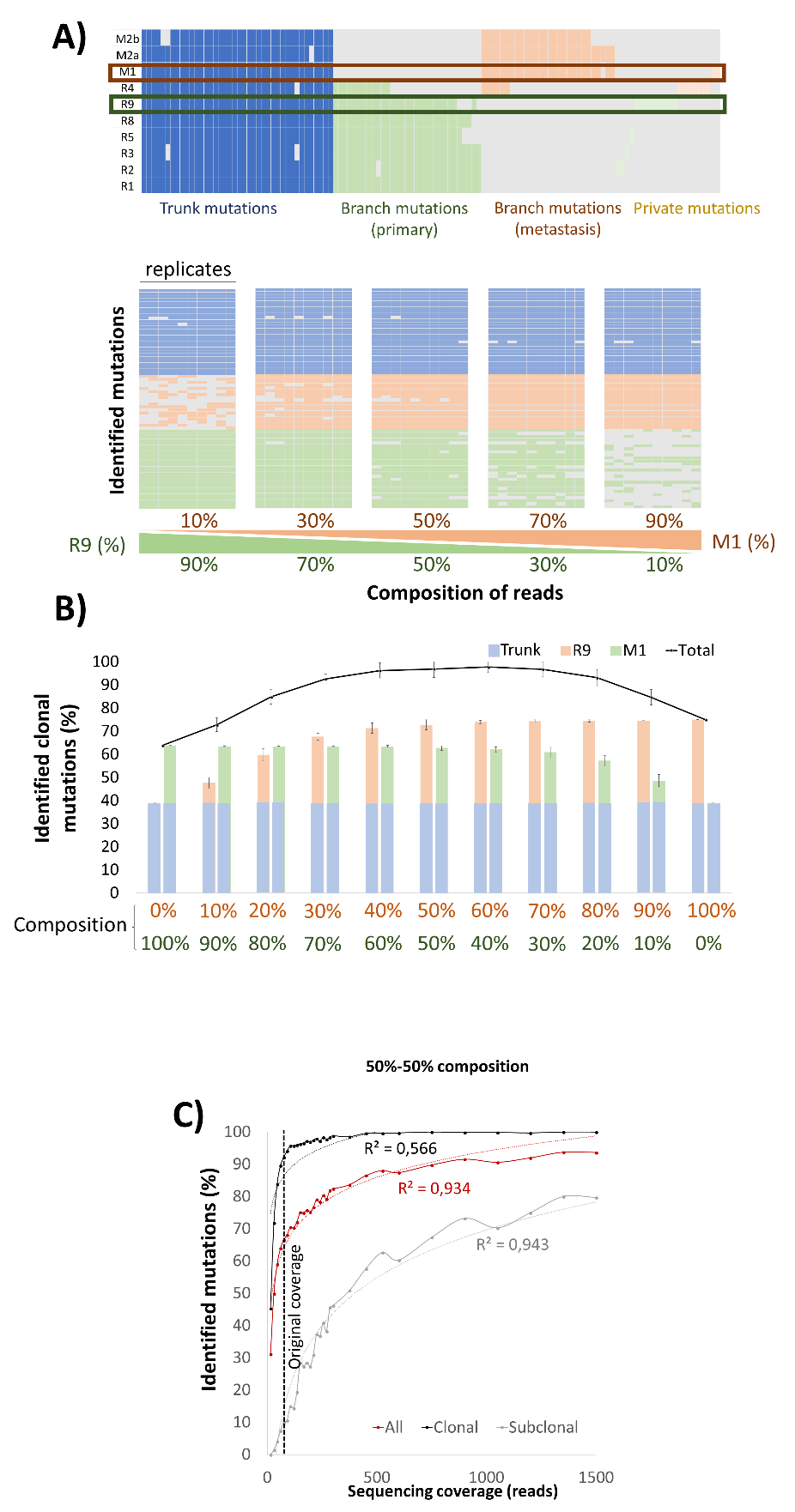

Supplement: Supplementary file 1 — Supplementary Figue 1. [file 41598_2020_71382_MOESM1_ESM.docx]
